# IRF3 and IRF8 Regulate NF-κB Signaling by Targeting MyD88 in Teleost Fish

**DOI:** 10.3389/fimmu.2020.00606

**Published:** 2020-04-16

**Authors:** Xiaolong Yan, Xueyan Zhao, Ruixuan Huo, Tianjun Xu

**Affiliations:** ^1^Laboratory of Fish Molecular Immunology, College of Fisheries and Life Science, Shanghai Ocean University, Shanghai, China; ^2^Laboratory for Marine Biology and Biotechnology, Qingdao National Laboratory for Marine Science and Technology, Qingdao, China; ^3^National Pathogen Collection Center for Aquatic Animals, Shanghai Ocean University, Shanghai, China; ^4^Key Laboratory of Exploration and Utilization of Aquatic Genetic Resources, Ministry of Education, Shanghai Ocean University, Shanghai, China; ^5^International Research Center for Marine Biosciences, Ministry of Science and Technology, Shanghai Ocean University, Shanghai, China

**Keywords:** MyD88, IRF3, IRF8, NF-κB, ubiquitination

## Abstract

MyD88 is a conserved intracellular adaptor, which plays an important role in the innate immune system. MyD88 transmits signals for downstream of toll-like and IL-1 receptors to activate NF-κB signaling pathway, which is tightly controlled in the immune response to maintain immune intensity and immune homeostasis at different stages. NF-κB signaling pathway has been extensively studied in mammals, but regulatory molecular mechanism is still unclear in teleost fish. We determined that IRF3 and IRF8 can regulate MyD88-mediated NF-κB signaling pathway in fish. Interestingly, MyD88 is precisely regulated by IRF3 and IRF8 through the same mechanism but in completely opposite ways. IRF3 promotes MyD88-mediated NF-κB signaling pathway, whereas IRF8 inhibits the signaling pathway. MyD88 is regulated via ubiquitin–proteasome degradation, whereas IRF3 or IRF8 inhibited or promoted MyD88 degradation in this pathway. Specifically, in the early stage of lipopolysaccharide (LPS) stimulation or *Vibrio* infection, up-regulation of IRF3 and down-regulation of IRF8 eventually increased MyD88 expression to activate the NF-κB signaling pathway to trigger immune response. In the late stage of stimulation, down-regulated IRF3 and up-regulated IRF8 synergistically regulate the expression of MyD88 to a normal level, thus maintaining the immune balance of homeostasis and preventing serious damage from persistent over-immunization. This study presents information on Myd88–NF-κB signaling pathway in teleost fish and provides new insights into its regulatory mechanism in fish immune system.

## Introduction

Innate immune system consists of germline-encoded pattern-recognition receptors (PRRs) that can identify microbial pathogens (1). Certain PRRs, such as toll-like receptors (TLRs), RIG-I-like receptors (RLRs), and NOD-like receptors (NLRs) have been discovered (2, 3). PRR-activated intracellular signaling pathways, such as NF-κB and IRF3, induce the production of inflammatory cytokine and interferon for defense against invading pathogens. TLRs are typical PRRs that are highly conserved in vertebrates and play an important role in innate immune system. As themost characterized PRRs, TLRs are type I transmembrane proteins that interact with downstream adapter proteins through intracellular toll/IL-1 receptor (TIR) domain (4, 5). TLRs are recognized by corresponding ligands and consequently activate downstream signaling pathways, including MyD88-dependent and independent pathways, which is also called TRIF-dependent pathway. After TRIF is recruited to TLR3 and TLR4, TRIF-dependent pathway is activated, leading to the activation of the NF-κB and IRF3 pathways (6–8). All TLRs in mammals, except TLR3, conduct signal transduction through the MyD88-dependent pathway, and these signals are transmitted via a series of molecules, such as IL-1R-associated kinase (IRAK) 1, IRAK4, and TNFR-associated factor 6 (TRAF6). Eventually, NF-κB is activated, thereby promoting the expression of inflammatory cytokines (9–12). Generally, immune signaling pathways are activated to trigger the immune response upon pathogen invasion. However, excessive immune responses lead to many inflammatory and autoimmune diseases (13). Therefore, to avoid over- immunity or insufficient immunity, organisms’ immune responses have evolved regulatory mechanisms for the regulation of immune response to maintain immune balance.

Toll-like receptors pathway is the most important innate immune signaling pathway that must be controlled to trigger the immune response or avoid excessive immune responses at different stages. So far, many regulatory factors participate in TLRs signaling pathway, including non-coding and coding genes (13). Among non-coding genes, microRNAs are involved in post-transcriptional regulation of TLR signaling pathways at different stages (3). For example, in a previous study, mycobacteria-induced miR-146a modulated inflammatory response by targeting 3′-untranslated regions (3′-UTR) of IRAK1 and TRAF6 and facilitated mycobacteria replication in macrophages (14). MyD88, an important adaptor molecule of TLRs signaling pathway, is negatively regulated by microRNAs (miR-155 and miR-200b/c) targeting Myd88 3′-UTR region directly to inhibit NF-κB activation and to reduce inflammation (15, 16). The mechanism of signal pathway regulation through protein-molecule interaction attracts many researchers’ attention, although non-coding genes regulate target molecules in various ways. For example, ADAM15 and TRIM38 inhibit NF-κB and TLR3-mediated type I interferon signaling pathways, respectively, by targeting TRIF (17, 18). NLRX1 can inhibit TLR-induced NF-κB signaling by interacting with TRAF6 and IKK, thereby affecting its susceptibility to lipopolysaccharide (LPS)-induced septic shock and plasma IL-6 level (19). In addition, interferon regulatory factors (IRFs) regulate the immune signal pathway aside from their inherent function. For example, IRF4 and IRF5 compete with MyD88 to negatively regulate TLR signaling pathway in mammals (20). However, there are no studies on IRFs that regulate MyD88-mediated downstream signaling pathway in fish. Overall, tight regulation of adaptor molecules and signaling proteins in TLRs signaling pathway is an essential part of immune regulatory mechanism.

Signal transduction of TLR pathway is highly conserved in invertebrates and mammals (21). As the main adaptor molecule in TLRs signaling pathway, the structure of MyD88 is conserved, its homologs have been identified in many vertebrate species (22, 23), and MyD88 has been extensively studied more than other TLR adaptors. However, lesser information about the function of MyD88 in lower vertebrates is available than in mammals (24, 25). In this study, we provide insights into the mechanisms that regulate MyD88-mediated NF-κB signaling pathway by targeting MyD88 in teleost fish, miiuy croaker (*Miichthys miiuy*), which is an excellent fish model for studying the mechanisms of some molecules in regulation of immune response (24–28). Specifically, after being stimulated by LPS or infected by *Vibrio anguillarum* or *Vibrio harveyi*, the expression of IRF3 and IRF8 in miiuy croaker changed dramatically, respectively. *V. anguillarum* and *V. harveyi* are the most typical Gram-negative pathogen for a wide range of marine animals and has been reported to cause high mortality throughout the world of aquaculture. In the early stage of LPS stimulation or *Vibrio* infection, IRF3 expression was up-regulated, which then showed a downward trend in the late stage of stimulation. On the contrary, IRF8 was down-regulated in the early stage and then up-regulated in the late stage of LPS stimulation or *Vibrio* infection. Further investigation revealed that by targeting MyD88, NF-κB signaling pathway is positively regulated by IRF3 and negatively regulated by IRF8. The knockdown of IRF3 can inhibit MyD88-mediated NF-κB signaling pathway, whereas its overexpression has the opposite effect. Conversely, IRF8 overexpression inhibited MyD88-mediated NF-κB signaling pathway, whereas its knockdown has the opposite effect. We further determined that inhibition and promotion of MyD88 degradation by IRF3 and IRF8, respectively, are both via the ubiquitin–proteasome pathway. In other words, in the early stage of initiating the immune response after *Vibrio* infection or LPS stimulation, up-regulation of IRF3 and down-regulation of IRF8 eventually increased MyD88 expression to activate the NF-κB signaling pathway to trigger immune response. Afterward, in the late stage of stimulation, down-regulated IRF3 and up-regulated IRF8 synergistically regulate the expression of MyD88 to a normal level, thus maintaining immune balance and preventing serious damage from persistent over-immunization. Overall, both IRF3 and IRF8 regulate the expression of MyD88 in opposite ways via the same ubiquitin–proteasome degradation. IRF3 and IRF8 can tightly regulate and stabilize MyD88-mediated NF-κB signaling pathway to maintain immune intensity and immune homeostasis at different stages. To our knowledge, this is the first report on the regulation of immune balance in fish coordinated by different members of the same gene family.

## Materials and Methods

### Samples and Challenge Experiments

Miiuy croakers were reared in seawater tanks at 25°C, and healthy individuals (∼50 g) were selected for pathogen infection experiment. Healthy fishes were randomly divided into different groups, namely, the control and injection groups. In the injection group, individual fish was kept in separate tanks and correspondingly injected with 0.1 ml of suspension of LPS (1 mg/ml, InvivoGen), 0.1 ml of *Vibrio harveyi* (3 × 10^8^ CFU/ml), and 0.1 ml of *Vibrio anguillarum* (3 × 10^8^ CFU/ml). Physiological water (0.1 ml) was used as control. After injection, fish were sacrificed at various time points (0, 3, 6, 12, 24, 36, 48, 72, and 96 h), and the liver was collected; at least three individuals were collected at each time point after stimulation. All animal experimental procedures were performed in accordance with the National Institutes of Health’s Guide for the Care and Use of Laboratory Animals, and the experimental protocols were approved by the Research Ethics Committee of Shanghai Ocean University (No. SHOU-DW-2018-047).

### Plasmid Construction

To construct the MyD88 expression vector, the open reading frame (ORF) of miiuy croaker MyD88 gene (GenBank accession number: JQ178357) was cloned from cDNA of miiuy croaker into *Kpn*I and *Xba*I sites of pcDNA3.1 (Invitrogen) with Myc tag and to *Kpn*I and *Bam*HI sites of pEGFP-N1 (Invitrogen). The ORF of miiuy croaker IRF3 (GenBank accession number: KF569501) gene was cloned from cDNA of miiuy croaker into *Kpn*I and *Xba*I sites of pcDNA3.1 with HA tag. The ORF of miiuy croaker IRF8 (GenBank accession number: KF569504) gene was cloned from cDNA of miiuy croaker into *Bam*HI and *Xba*I sites of pcDNA3.1 with Flag tag. IRF3 mutations, including IRF3ΔDBD (ΔDBD), IRF3ΔSRD (ΔSRD), and IRF3ΔIAD (ΔIAD), were generated by PCR using specific primers on the basis of IRF3 recombinant plasmid. IRF3-shRNA was designed and ligated into *Bam*HI and *Eco*RI of pSIREN-RetroQZsGreen1 vector (Clontech). IRF8 mutations, including IRF8ΔIRF (ΔIRF) and IRF8ΔIRF3 (ΔIRF3), were generated by PCR using specific primers on the basis of IRF8 recombinant plasmid. IRF8-shRNA was designed and ligated into *Bam*HI and *Eco*RI of pSIREN-RetroQZsGreen1 vector. The pRK5-HA-Ubiquitin-WT (ubiquitin–HA) plasmid was purchased from Addgene. All recombinant plasmids were affirmed by DNA sequencing. All plasmids were extracted using EndotoxinFree Plasmid DNA Miniprep Kit (Tiangen). Primer sequences were listed in Supplementary Table S1.

### Cell Culture and Transient Transfections

Fish epithelioma papulosum cyprini (EPC) cells were maintained in medium 199 (HyClone) supplemented with 10% fetal bovine serum (FBS; Gibco), 2 mM of L-glutamine, 100 U/ml of penicillin, and 100 mg/ml of streptomycin under humidified conditions at 26°C with 5% CO_2_. Human embryonic kidney (HEK) 293 cells were maintained in Dulbecco’s modified Eagle medium (DMEM) (HyClone) supplemented with 10% FBS, 2 mM of L-glutamine, 100 U/ml of penicillin, and 100 mg/ml of streptomycin under humidified conditions at 37°C with 5% CO_2_. EPC and HEK293 cells were transfected with various plasmids using Lipofectamine 3000^TM^ (Invitrogen). In addition, proteasome inhibitor MG132 (sigma) or cycloheximide (CHX) (Beyotime) was added into the medium at 24 h post transfection and used at a final concentration of 30 μM/ml and 100 μg/ml (29).

### RNA Isolation and qRT-PCR Analysis

Total RNA was isolated with TRIzol reagent (TaKaRa) according to the manufacturer’s instructions. First-strand cDNA was synthesized using FastQuant RT Kit (Tiangen), which included DNase treatment of RNA to eliminate genomic contamination. qRT-PCR was performed using SYBR Premix Ex Taq Kit (TaKaRa) on QuantStudio 3 real-time PCR system (Applied Biosystems, United States). PCR cycling conditions were as follows: 10 s at 95°C, followed by 40 cycles of 5 s at 95°C, and then 31 s at 60°C (30). All primers used for qRT-PCR are shown in Supplementary Table S1. β-Actin and EF-1α were used as internal control for double checking. Three independent experiments were conducted for statistical analysis. The gene-specific primer sequences were listed in Supplementary Table S1.

### Luciferase Reporter Assay

Expression plasmids and reporter gene plasmids including NF-κB, IL-1β, and IL-8 (31) were transfected in EPC cells, and *Renilla* luciferase reporter plasmid (pRL-TK, Promega) was regarded as internal control. The ratio of pRL-TK to reporter gene plasmids was 1:10. Control group was compared with experimental group by adding equal amount of relevant empty vectors. Reporter luciferase activities were measured using Dual-Luciferase reporter assay system (Promega). Each experiment was conducted three times independently, and the results were obtained (32).

### Immunoblot Assay

HEK293 or EPC cells were washed three times using sterile and cold phosphate-buffered saline (PBS). Then, cells were lysed by cell lysis buffer [20 mM of Tris (pH 7.5), 150 mM of NaCl, and 1% Triton X-100]. Protein concentrations were measured via bicinchoninic acid (BCA) assay (Pierce) and equalized with extraction reagent. Equal amounts of extracts were mixed with 2 × sodium dodecyl sulfate (SDS) loading buffer, loaded onto SDS–polyacrylamide gel electrophoresis (PAGE), and then transferred onto polyvinylidene difluoride (PVDF) membranes (Millipore) using semi-dry blotting system (Bio-Rad Trans Blot Turbo System). The membranes were washed thrice using TBST buffer (20 mM of Tris, 150 mM of NaCl, and 0.1% Tween 20, pH 7.5) and blocked at room temperature using 5% dried skimmed milk by TBST diluted on rocker platform for 90 min. The membranes were then incubated with primary antibodies at 4°C overnight. Primary antibodies used in this study were against Myc, HA, Flag, and Tubulin (Abcam). The membranes were washed thrice using TBST and incubated with secondary antibody at room temperature on the rocker platform for 60 min. Finally, immunoreactive proteins were detected with WesternBright^TM^ ECL (Advansta), and digital imaging was performed by cold charged coupled device (CCD) camera.

### Immunoprecipitation Assay

For immunoprecipitation (IP) experiments, 5 μg of total plasmids were cotransfected into HEK293 cells, which were cultured into 10-cm^2^ plate overnight. After 48 h from transfection, the cells were washed thrice with cold PBS. The cells were lysed with western and IP lysis buffer containing protease inhibitor cocktail (Bitake) and phenylmethylsulfonyl fluoride (PMSF) (Beyotime) at 4°C for 20 min on a rocker platform. The cellular fragment was separated by centrifugation at 12,000 *g* for 10 min at 4°C. After centrifugation, the supernatant was transferred into a new centrifuge tube and incubated with protein A + G (Sigma) and monoclonal anti-Myc (Abcam) overnight at 4°C with soft agitation. The following day, IP protein was collected by centrifugation at 2,500 *g* for 5 min at 4°C. Then, the beads were washed three times with western and IP lysis buffer and resuspended in 60 μl of 2 × SDS loading buffer. Immunoprecipitates and whole cell lysates were analyzed by immunoblotting.

### Fluorescent Microscopy

HEK293 cells were seeded onto 24-well plates and transfected with indicated plasmids for 48 h using Lipofectamine^TM^ 3000 (Invitrogen). Then, images were obtained under a fluorescence microscope (Leica).

### RNA Interference

Miiuy croaker IRF3-specific siRNA (si-IRF3) and IRF8-specific siRNA (si-IRF8) were 5′-GCUUCAAACUGGUCUCUGATT-3′ (sense), 5′-UCAGAGACCAGUUUGAAGCTT-3′ (antisense) and 5′-GCCGCACUUUGUUUCGAAUTT-3′ (sense), 5′-AUUCGAAACAAAGUGCGGCTT-3′ (antisense), respectively. The scrambled control RNA (si-Ctrl) sequences were 5′-UUCUCCGAACGUGUCACGUTT-3′ (sense) and 5′-ACGUGACACGUUCGGAGAATT-3′ (antisense). The si-IRF3 and si-IRF8 can specifically identify the miiuy croaker IRF3 and IRF8 gene. EPC cells were transfected with 100 nM of siRNA using Lipofectamine 2000^TM^ (Invitrogen).

### Statistical Analysis

All experiments were performed in at least three independent experiments with three technical replicates per experiment. Relative gene expression data were obtained using the 2^ΔΔCT^ method, and comparisons between groups were analyzed by one-way analysis of variance followed by Duncan’s multiple comparison tests. Results are expressed as mean ± SE (standard error), and significant differences between groups were determined by a two-tailed Student *t*-test (33).

## Results

### IRF3 and IRF8 Are Regulated After Lipopolysaccharide and *Vibrio* Induction

**FIGURE 1:**
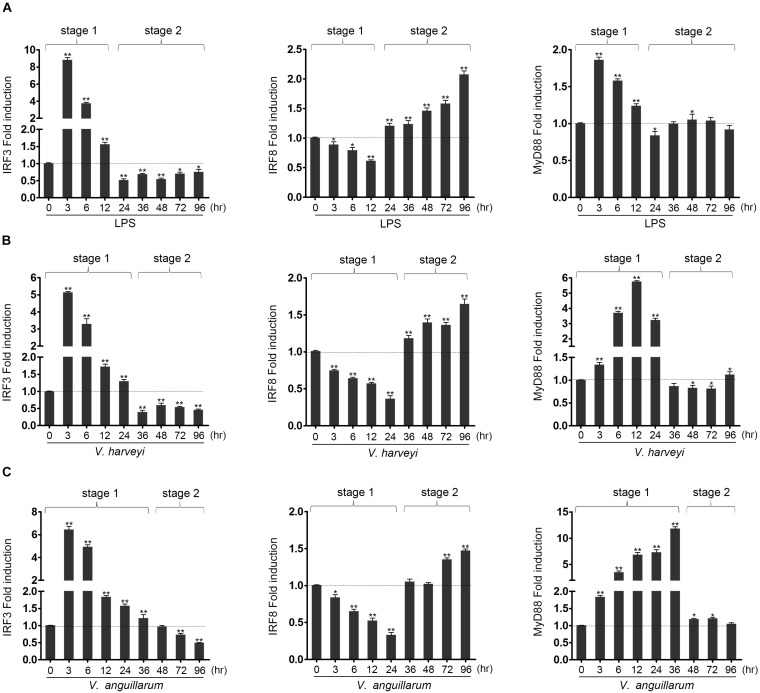
The expression profiles of IRF3, IRF8 and MyD88 in liver after lipopolysaccharide (LPS) and *Vibrio* induction. The expression profiles of IRF3, IRF8, and MyD88 has been analyzed in liver at 3, 6, 12, 24, 36, 48, 72, and 96 h after induction with LPS **(A)**, as well as infected with *Vibrio harveyi* **(B)** or *Vibrio anguillarum* **(C)**. The total RNAs were extracted, and the expression of IRF3, IRF8, and MyD88 was examined by qRT-PCR. All experiments were performed in at least three independent experiments. *p < 0.05 and **p < 0.01 versus the controls.

Miiuy croaker individuals were infected with *Vibrio harveyi* or *Vibrio anguillarum* or stimulated with LPS to determine whether the expression of hosts IRF3, IRF8, and MyD88 was regulated by pathogen stimulation. The expressions of these genes were detected by qRT-PCR. With regard to the whole stress process of LPS stimulation or *Vibrio* infection, it could be divided into two distinct stages – stage 1 and stage 2 – as shown in Figure 1. In the first stage, the expression of IRF3 was up-regulated compared with that of control group, whereas the expression of IRF8 was down-regulated. The expression of Myd88 was up-regulated rapidly. In the second stage, the expression of IRF3 was down-regulated compared with that of control group, whereas the expression of IRF8 was up-regulated. In this stage, the expression of MyD88 remained relatively stable. The difference is that the effects of the three stimuli on gene expression differ in the duration of the stage 1 and stage 2, which may be due to the immune characteristics of different pathogens. The above results show the amount of expression of the IRF3, IRF8, and MyD88 after LPS stimulation and *Vibrio* infection. These results imply that IRF3 and IRF8 may play an important role in the immune response after LPS stimulation and *Vibrio* infection.

### IRF3 and IRF8 Regulate the MyD88-Mediated NF-κB Signaling Pathway

**FIGURE 2:**
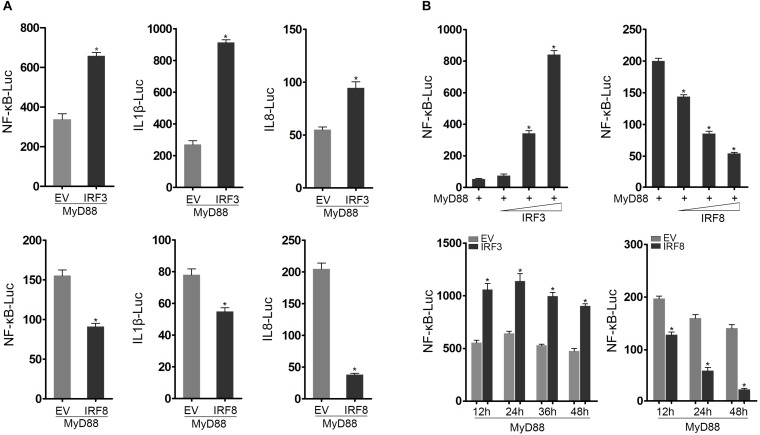
IRF3 and IRF8 regulate MyD88-mediated NF-κB signaling pathway. **(A)** Epithelioma papulosum cyprini (EPC) cells were seeded in 24-well plates and cotransfected with 0.2 μg of MyD88 and 0.2 μg of pcDNA3.1, IRF3 or IRF8, respectively, together with 0.25 μg of NF-κB, IL-1β reporter gene or IL-8 reporter gene, respectively. After 24 h post transfection, the luciferase activity was measured. **(B)** The concentration gradient experiment of IRF3 (0.05, 0.1, or 0.2 μg) or IRF8 (0.05, 0.1, or 0.2 μg) expression plasmid within 0.2 μg of MyD88 and 0.25 μg of NF-κB reporter gene was conducted. After 24 h post transfection, the luciferase activity was measured. After being cotransfected with MyD88 and IRF3 or IRF8 expression plasmids, together with NF-κB reporter gene, the luciferase activity was measured at different time points. The “EV” represents pcDNA3.1 empty plasmid. The luciferase activity value was achieved against the *Renilla* luciferase activity. *p < 0.05 versus the controls. All experiments were performed in at least three independent experiments.

Using luciferase reporter assays, we initially found that MyD88-mediated NF-κB signaling pathway was promoted by IRF3 and inhibited by IRF8 compared with the control. In addition, cotransfection of EPC cells with IRF3 and MyD88 promoted IL-1β and IL-8 reporter genes, whereas cotransfection of EPC cells with IRF8 and MyD88 suppressed IL-1β and IL-8 activation (Figure 2A). We further examined the IRF3 and IRF8 concentration gradient in EPC cells at different time points to explore the regulation effect of IRF3 and IRF8 (Figure 2B). Data showed that MyD88-mediated NF-κB signaling pathway was promoted by IRF3 and inhibited by IRF8.

### IRF3 and IRF8 Regulate the MyD88 Expression

**FIGURE 3:**
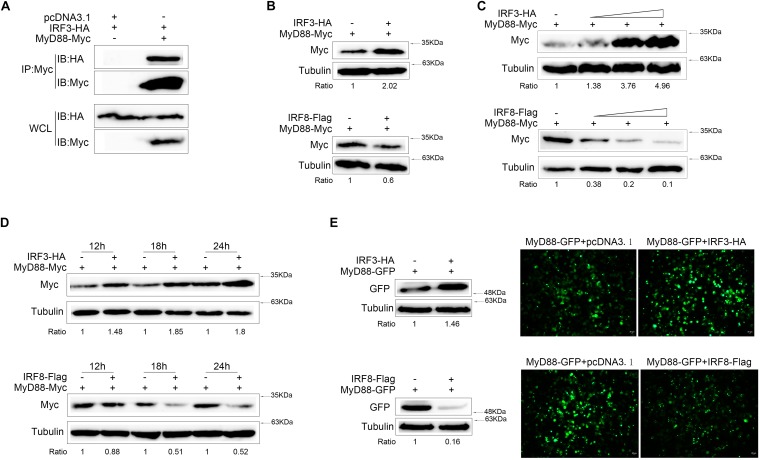
MyD88 expression was regulated by IRF3 and IRF8. **(A)** HEK293 cells were seeded in 10-cm^2^ dishes and cotransfected with 2 μg of pcDNA3.1 or 2 μg of IRF3 together with 3 μg of MyD88. After 24 h post transfection, cell lysates were immunoprecipitation (IP) with Myc antibody, and then immunoprecipitates and whole-cell lysate (WCL) were analyzed by immunoblot with the Abs indicated. **(B)** Epithelioma papulosum cyprini (EPC) cells were seeded in 12-well plates and transfected with 0.4 μg of MyD88-Myc together with 0.4 μg of pcDNA3.1, IRF3-HA, or IRF8-Flag for 24 h. Then, MyD88 was concluded by immunoblot assays and normalized to Tubulin. **(C)** The concentration experiment of 0.4 μg of pcDNA3.1, IRF3 (0.1, 0.2, or 0.4 μg), or IRF8 (0.1, 0.2, or 0.4 μg) plasmid together with 0.4 μg of MyD88 was conducted in EPC cells, and MyD88 was concluded by immunoblot assays. **(D)** The time gradient experiment of 0.4 μg of pcDNA3.1, IRF3, or IRF8 plasmid together with 0.4 μg of MyD88 was conducted in EPC cells, and MyD88 was concluded by immunoblot assays. **(E)** HEK293 cells were seeded in 12-well plates and cotransfected the 0.4 μg of IRF3 or IRF8 plasmid with 0.4 μg of MyD88-GFP. After 24 h post transfection, the fluorescence signals of MyD88-GFP were detected by fluorescence microscopy (right), and then MyD88 was determined by immunoblot assays (left). All experiments were performed in at least three independent experiments.

To investigate the interaction of IRF3 with MyD88 at protein level, IRF3-HA plasmid and MyD88-Myc plasmid were cotransfected into HEK293 cells. Anti-Myc antibody immunoprecipitated protein complexes containing MyD88 protein were recognized by anti-HA, thereby indicating that IRF3 protein was associated with MyD88 (Figure 3A). In addition, IRF3 or IRF8 and MyD88 were cotransfected into EPC cells, respectively, after 24 h from transfection, and MyD88 expression was examined by immunoblot assays. The level of MyD88 expression increased with increased levels of IRF3 expression and decreased levels of IRF8 expression (Figure 3B). On the basis of the above results, we examined a concentration gradient (Figure 3C) and time point experiments (Figure 3D). MyD88 was reduced in a dose-dependent manner. Then, MyD88-GFP expression plasmids and IRF3 or IRF8 were cotransfected into HEK293 cells and compared with the empty vector. As shown in the results, the fluorescence signals of MyD88-GFP markedly increased with increased levels of IRF3 expression and decreased levels of IRF8 expression, compared with the empty vector (Figure 3E). The cells were lysed, and MyD88 protein was detected with GFP antibody by immunoblot assays. The expression of MyD88 was promoted by IRF3 and inhibited by IRF8.

### Effects of IRF3 or IRF8 Knockdown on MyD88-Mediated NF-κB Activation

**FIGURE 4:**
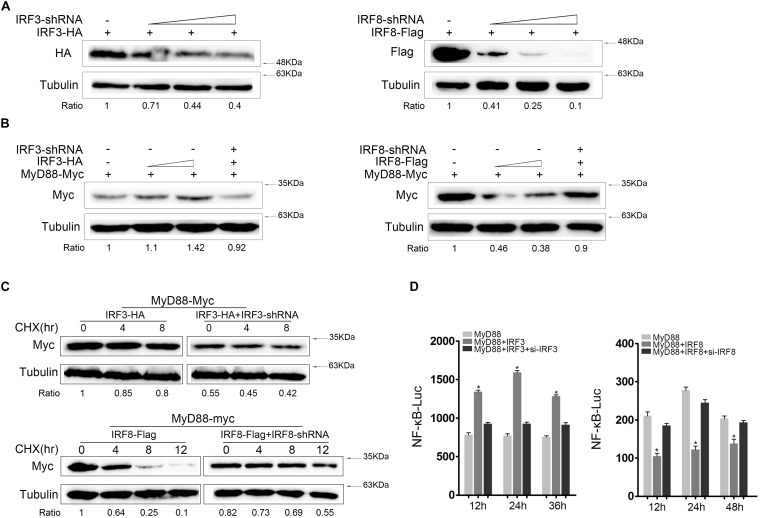
Knockdown of IRF3 or IRF8 regulate MyD88-mediated NF-κB activation. **(A)** HEK293 cells were cotransfected with the 0.4 μg of IRF3 or 0.4 μg of IRF8 plasmids with different concentrations of IRF3-shRNA (0.1, 0.2, or 0.4 μg) or IRF8-shRNA (0.1, 0.2, or 0.4 μg), respectively. After 24 h post transfection, IRF3 or IRF8 was determined by immunoblot assays. **(B)** HEK293 cells were seeded in 12-well plates and cotransfected with the IRF3 (0.2 or 0.4 μg) or IRF8 (0.2 or 0.4 μg) plasmids together with 0.4 μg of MyD88 and then 0.4 μg of IRF3 or IRF8 plasmid in the fourth well with 0.4 μg of IRF3-shRNA or IRF8-shRNA, respectively, together with 0.4 μg of MyD88. MyD88 was determined by immunoblot assays. **(C)** HEK293 cells were seeded in 12-well plates and cotransfected with 0.4 μg of IRF3 and IRF3-shRNA plasmids or IRF8 and IRF8-shRNA plasmids, respectively, together with 0.4 μg of MyD88. At 24 h post transfection, and cells were treated with CHX (100 μg/ml) and lysed at different time points. The expression of MyD88 was examined by immunoblot assays. **(D)** EPC cells were seeded in 24-well plates and cotransfected with 0.2 μg of IRF3 plasmid and IRF3-siRNA or 0.2 μg of IRF8 plasmid and IRF8-siRNA, respectively, together with 0.1 μg of MyD88 and 0.25 μg of NF-κB reporter gene, and the luciferase activity was measured at different times.*p < 0.05 versus the controls. All experiments were performed in at least three independent experiments.

MyD88 expression was increased in the presence of IRF3 and decreased in the presence of IRF8. Therefore, we tried to knockdown IRF3 or IRF8 to verify its effect on MyD88 expression. We constructed the IRF3-shRNA and IRF8-shRNA knockdown plasmids. IRF3 or IRF8 expression decreases with increasing IRF3-shRNA or IRF8-shRNA concentration. We confirmed that IRF3-shRNA and IRF8-shRNA plasmids efficiently down-regulated IRF3 and IRF8 expressions (Figure 4A). HEK293 cells were cotransfected with MyD88, IRF3, and IRF3-shRNA or MyD88, IRF8, and IRF8-shRNA plasmids; and MyD88 expression was examined using immunoblot assays. According to the results, MyD88 expression increased with increasing IRF3 plasmid concentration and decreased with increasing IRF8 plasmid concentration (Figure 4B). At 24 h post transfection, the cells were treated with CHX reagent before lysing to inhibit protein translation, and MyD88 expression was examined by immunoblot assays. As shown in the results, IRF3-shRNA also inhibited IRF3 and increased MyD88 degradation, whereas IRF8 had the opposite effect (Figure 4C). To demonstrate the regulation effect of IRF3 and IRF8 on MyD88-mediated NF-κB pathway, IRF3 plasmid and IRF3-siRNA or IRF8 plasmid and IRF8-siRNA were cotransfected into HEK293 cells together with MyD88, and a time gradient experiment was conducted to confirm the above results. Data showed that the promoting effect of IRF3 and the inhibitory effect of IRF8 were constant at different time points (Figure 4D).

### IRF3-DBD and IRF8-IRF Domain Is the Core Regulatory Domain

**FIGURE 5:**
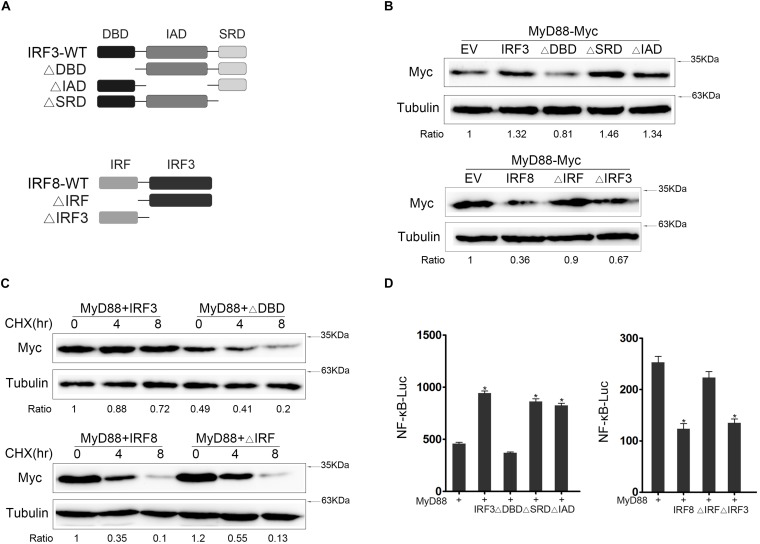
The core regulatory domain of IRF3 and IRF8. **(A)** Schematic diagram of the wild type (WT) and mutants of IRF3 or IRF8. **(B)** Epithelioma papulosum cyprini (EPC) cells were seeded in 12-well plates and transfected with 0.4 μg of IRF3 and IRF3 mutant plasmids with 0.4 μg of MyD88. At 24 h post transfection, the immunoblot assays were measured. EPC cells were seeded in 12-well plates and transfected with 0.4 μg of IRF8 plasmid and IRF8 mutant plasmids with 0.4 μg of MyD88 plasmid. At 24 h post transfection, the immunoblot assays were measured. **(C)** HEK293 cells were seeded in 12-well plates and transfected 0.4 μg of IRF3 and ΔDBD plasmids or IRF8 and IRF8ΔIRF together with 0.4 μg of MyD88. At 24 h post transfection, the cells were treated with CHX (100 μg/ml) for different times, and then MyD88 was determined by immunoblot assays. **(D)** EPC cells were seeded in 24-well plates and cotransfected with 0.2 μg of IRF3 and IRF3 mutant plasmids or IRF8 and IRF8 mutant plasmids, respectively, together with 0.2 μg of MyD88 and 0.25 μg of NF-κB reporter gene, and the luciferase activity was measured. *p < 0.05 versus the controls. All experiments were performed in at least three independent experiments.

Three mutants of IRF3 [namely, IRF3ΔIAD (ΔIAD), IRF3ΔDBD (ΔDBD), and IRF3ΔSRD (ΔSRD)] and two mutants of IRF8 [namely, IRF8ΔIRF (ΔIRF) and IRF8ΔIRF3 (ΔIRF3)] were constructed (Figure 5A) to further confirm which functional domain of IRF3 and IRF8 affected MyD88 expression. We tested the interaction domain of IRF3 or IRF8 associated with MyD88 in EPC cells. The results showed that DBD domain of IRF3 and IRF domain of IRF8 was the core regulatory domain affecting MyD88 expression (Figure 5B). IRF3 and IRF3ΔDBD plasmids or IRF8 and IRF8ΔIRF plasmids were cotransfected with MyD88; at 24 h post transfection, the cells were treated with CHX before lysing to inhibit protein translation, and MyD88 expression was examined by immunoblot assays. Data showed that the promoting effect of IRF3 primarily depends on DBD domain and the inhibitory effect of IRF8 primarily depends on IRF domain (Figure 5C). Then, we confirmed the regulation effect of IRF3 and mutants of IRF3 or IRF8 and mutants of IRF8 on MyD88-mediated NF-κB pathway using Luciferase reporter assays (Figure 5D). IRF3 affected MyD88 expression through the DBD domain, thereby inhibiting MyD88 degradation. Furthermore, IRF domain is the primary domain of IRF8 that promoted MyD88 degradation.

### IRF3 and IRF8 Regulate MyD88 Degradation Through Proteasome Pathway

**FIGURE 6:**
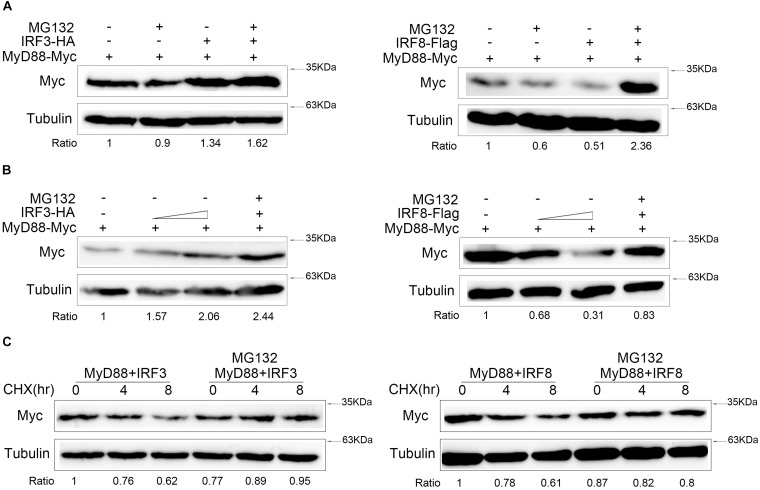
IRF3 and IRF8 regulate MyD88 degradation through ubiquitin–proteasome pathway. **(A)** HEK293 cells were seeded in 12-well plates and transfected with 0.4 μg of MyD88 and IRF3 plasmids or IRF8 plasmids for 24 h, and then the cells were treated with dimethyl sulfoxide (DMSO) or 30 μM of MG132 for 10 h. MyD88 was determined by immunoblot assays. **(B)** HEK293 cells were transfected with 0.4 μg of MyD88 and concentration gradient of IRF3 plasmids (0.2 or 0.4 μg) or concentration gradient of IRF8 plasmids (0.2 or 0.4 μg). At 24 h post transfection, the cells were treated with DMSO or 30 μM of MG132 for 12 h, and MyD88 was determined by immunoblot assays. **(C)** HEK293 cells were transfected with 0.4 μg of MyD88 and IRF3 plasmids or IRF8 plasmids. After 24-h transfection, the cells were treated with CHX (100 μg/ml), and the experiment group was treated with 30 μM of MG132 for different times. MyD88 was determined by immunoblot assays. All experiments were performed in at least three independent experiments.

Generally, protein degradation involved three pathways, namely, ubiquitin–proteasome, lysosomal, and autophagosome pathways. To confirm which pathway mediated IRF3 and IRF8 regulate MyD88 degradation, we cotransfected with MyD88 and IRF3 or IRF8 plasmids, and the cells were treated with indicated inhibitors MG132 reagent (Figure 6A). MyD88 degradation was inhibited in a dose-dependent manner with increasing IRF3 plasmid, and MyD88 can be further rescued after MG132 treatment. The down-regulation of MyD88 by IRF8 was also rescued by MG132 dose dependently (Figure 6B). After 24 h post transfection with MyD88 and IRF3 or IRF8 plasmids, the cells were treated with CHX. Meanwhile, experimental group cells were treated with MG132, and these results showed that MyD88 degradation was inhibited by IRF3 and promoted by IRF8 (Figure 6C).

### IRF3 and IRF8 Have the Opposite Effects on MyD88 Polyubiquitination and Its Half-Life

**FIGURE 7:**
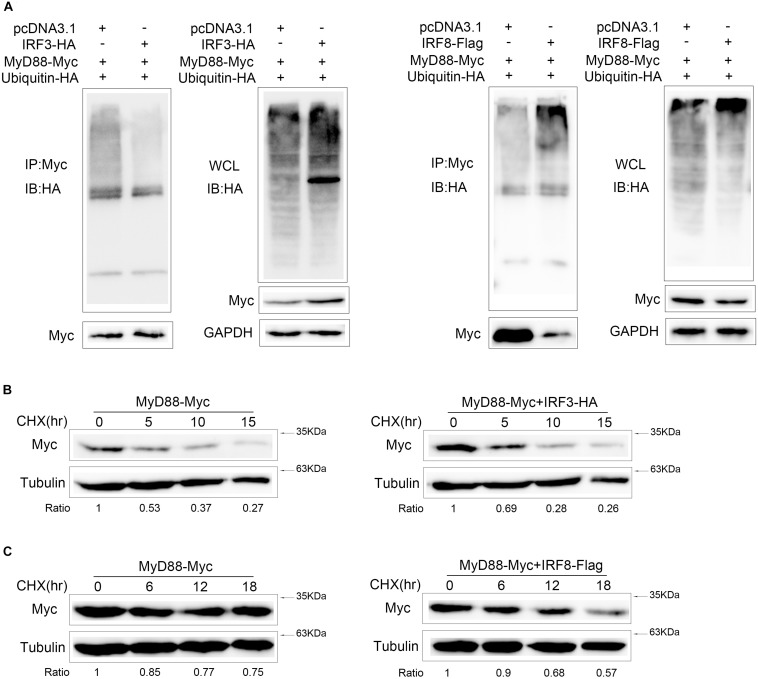
IRF3 and IRF8 have the opposite effects on MyD88 polyubiquitination and its half-life. **(A)** HEK293 cells were seeded in 10-cm^2^ dishes and cotransfected with 2 μg of IRF3 or IRF8 and ubiquitin–HA plasmids together with 3 μg of MyD88. At 24 h post transfection, the cells were treated with MG132 for 10 h. Then, the cells were lysed and IP analyses with Myc antibody and then WB with antibody against HA. Samples of whole-cell lysate (WCL) were included as controls. **(B)** Epithelioma papulosum cyprini (EPC) cells were transiently cotransfected with 0.4 μg of MyD88 and IRF3 plasmids for 24 h, and then the cells were treated with CHX (100 μg/ml) and harvested at different time points. MyD88 was determined by immunoblot assays. **(C)** EPC cells were cotransfected with 0.4 μg of MyD88 and IRF8 plasmids for 24 h, and the cells were treated with CHX (100 μg/ml) and harvested at different time points. MyD88 was determined by immunoblot assays. All experiments were performed in at least three independent experiments.

Proteasome degradation process of Myd88 was inhibited by IRF3 and promoted by IRF8. To further confirm that the MyD88 degradation depends on ubiquitin–proteasome pathway, ubiquitination plasmid together with MyD88 and IRF3 or IRF8 plasmids was cotransfected into HEK293 cells. Then, the cells were lysed for IP with an antibody against Myc. Results of immunoblot assays with HA antibody showed that ubiquitinated MyD88 with IRF3 in the cells decreased to a greater extent than those in the cells transfected with an empty vector. Then, ubiquitination plasmid together with MyD88 and IRF8 was cotransfected into HEK293 cells. Results of immunoblot assays with HA antibody showed that IRF8 promoted the ubiquitination of MyD88 (Figure 7A). IRF3 and IRF8 regulated MyD88 polyubiquitination in the cells. Then, after 24 h post transfection of EPC cells with MyD88 and IRF3 plasmids, the cells were treated with CHX and lysed at different time points; immunoblotting was subsequently performed. MyD88 expression increased at a higher rate than that in the cells transfected with empty vector (Figure 7B). After 24 h post transfection with MyD88 and IRF8 plasmids, the cells were treated with CHX and lysed at different time points. Then, immunoblotting was subsequently performed. MyD88 level reduced at a higher rate than that in the cells transfected with empty vector (Figure 7C).

## Discussion

In the innate immune response, NF-κB is an important transcription factor that mediates the production of numerous pro-inflammatory cytokines and plays a crucial role in many signaling pathways (34). Therefore, tight regulation of NF-κB signaling pathway was crucial to maintain the balance of immunity homeostasis. Many studies have reported certain ways to regulate NF-κB activity. For instance, pro-inflammatory cytokines TNFα and IL-6 could induce TRIM52 expression, and TRIM52 as a positive regulator of NF-κB pathway, thereby forming a positive feedback regulation loop (35). WWP2 and TRIM38 can negatively regulate TLR-induced NF-κB signaling pathway by targeting different genes for ubiquitination and degradation (36, 37). Moreover, MARCH8 can negatively regulate IL-1β-induced NF-κB activation by targeting IL1RAP for ubiquitination and degradation (38). In fish, we have reported that miR-3570 and miR-214 can directly target the 3′-UTR region of MyD88 to affect NF-κB signaling pathway through post-transcriptional regulation (24, 25). In addition, some miRNAs were also found to regulate the NF-κB pathway by directly inhibiting the expression of NF-κB subunit p65 and IRAK4 (31, 39, 40). In this study, we confirmed that MyD88-mediated NF-κB signaling pathway was positively regulated by IRF3 and negatively regulated by IRF8 by targeting MyD88. IRF3 and IRF8 are capable of tightly regulation and stabilization of MyD88-mediated NF-κB signaling pathway in teleost fish through pathogen-induced expression of host IRF3 and IRF8.

More evidences indicate that IRF family members regulate the expressions of IFN genes and participate in the regulation of signaling pathways in innate immune (41). For example, IRF4 can negatively regulate of TLR signaling by targeting MyD88 (20) and IRF8 interacts with TRAF6 to participate in the MyD88-dependent activation of NF-κB to induce pro-inflammatory cytokines in mammals (42). Interestingly, previous studies have reported that fish (Atlantic salmon) MyD88 interacted with IRF3 and was involved in the positive regulation of IRF-induced IFN response (43). IRF3 can positively regulate NF-κB pathway, according to our results. This is the first time that IRF3 has been found to regulate Myd88-mediated NF-κB signaling pathway, thereby indicating that IRF3 plays an important regulatory role in different immune signaling pathways in fish. According to a previous research, interaction of different IRFs with target molecules was regarded as an important regulatory mechanism, and we found that IRF3 could interact with Myd88. Moreover, in this study, MyD88-mediated NF-κB signaling pathway was positively regulated by IRF3 and negatively regulated by IRF8 and further found that IRF3 and IRF8 maintain the stabilization of MyD88-mediated NF-κB signaling pathway through ubiquitin–proteasomal degradation in two completely opposite ways.

IRF3 contains three domains, namely, N-terminal DNA-binding domain (DBD), C-terminal IRF association domain (IAD), and another domain (SRD). Certain research reported that IRFs interact with other genes through different domains (44). For example, IAD could be involved in interaction with other IRFs except for IRF1 and IRF2 (45), and DBD domain is the core unit that is responsible for binding to the IRF element (46, 47). These results showed that wild IRF3 can inhibit the degradation of MyD88, whereas IRF3ΔDBD did not. The effects of IRF3 in inhibiting MyD88 degradation were weakened when DBD was knocked down. IRF8, which belongs to IRF4 subfamily, contains two domains, IRF domain and IRF3 domain. The IRF domain can interact selectively and non-covalently with a DNA region that regulates the transcription of a region of DNA (48). The IRF3 domain can interact with DNA sequence within the regulatory region of a gene to modulate transcription. In this study, we find that IRF domain may is the main domain for the interaction of IRF8 with MyD88. A series of studies have reported that Myd88 also interacted with other regulators, indicating that important genes are often regulated via different regulatory ways to achieve a stable immune balance. Myd88 is the adaptor molecule of all TLRs (except TLR3), and the regulation of Myd88 is the most important and effective way to maintain immune balance. Therefore, a variety of interactive ways to regulate Myd88 expression must be elucidated (47). In this study, we have found two completely opposite ways to achieve immune balance.

MyD88 plays an important role in immune response, and almost all TLRs conduct signal transduction through MyD88 except TLR3. After recognizing the PAMPs, TLRs recruits MyD88 to transduce the signals, and then NF-κB is activated (49, 50). Therefore, direct regulation of MyD88 may be the most effective method of regulating TLRs signaling pathway. For instance, some non-coding RNAs, such as miRNAs like miR-155 (15), miR-200b/c (16), miR-203 (51), miR-214 (25), and miR-3570 (24), have been reported to negatively regulate TLR-induced NF-κB activation by targeting MyD88. Other coding genes, such as IRF5, were generally involved in the downstream of TLR-MyD88 signaling pathway through interaction with MyD88 (52). We demonstrated for the first time that MyD88-mediated NF-κB signaling pathway was positively regulated by IRF3 and negatively regulated by IRF8 in teleost fish. In addition, IRF3 and IRF8 regulated MyD88 degradation through ubiquitin–proteasomal degradation pathway. In general, IRF3 and IRF8 of the IRFs family cooperatively maintained the stabilization of MyD88-mediated NF-κB signaling pathway through ubiquitin–proteasomal degradation pathway in two completely opposite ways.

In conclusion, MyD88 degradation was inhibited by IRF3 and promoted by IRF8 via the ubiquitin–proteasome pathway in teleost fish. In the first stage after *Vibrio* infection or LPS stimulation, up-regulation of IRF3 and down-regulation of IRF8 eventually increased MyD88 expression to activate the NF-κB signaling pathway to trigger immune response. In the second stage, down-regulated IRF3 and up-regulated IRF8 synergistically regulate the expression of MyD88 to a normal level to maintain the balance of immunity homeostasis. MyD88-mediated NF-κB signaling pathway is precisely regulated by IRF3 and IRF8 through the same mechanism but in completely opposite ways. These data present more information on Myd88–NF-κB signaling pathway in teleost fish and provide new insights into the different genes of the same family that cooperatively maintain the stabilization of immune signaling pathway through the same regulatory mechanism in different regulatory ways.

## Data Availability Statement

All datasets generated for this study are included in the article/Supplementary Material.

## Ethics Statement

The animal study was reviewed and approved by the Research Ethics Committee of Shanghai Ocean University (No. SHOU-DW-2018-047).

## Author Contributions

TX conceived and designed the experiments. TX, XY, XZ, and RH performed the experiments, analyzed the data, and contributed to reagents, materials, and analysis tools. TX and XY wrote the manuscript.

## Conflict of Interest

The authors declare that the research was conducted in the absence of any commercial or financial relationships that could be construed as a potential conflict of interest.
