# Supplementary material for: IRF3 and IRF8 Regulate NF-κB Signaling by Targeting MyD88 in Teleost Fish
Source: Front Immunol. 2020 Apr 16;11:606. doi: 10.3389/fimmu.2020.00606 (PMC7179762; doi:10.3389/fimmu.2020.00606)
Supplement: Supplementary file 1 [file Table_1.DOC]

**Supplemental Table 1.** PCR primer information in this study**.**

| **Primers** | **Sequences (5’-3’)** |
| --- | --- |
| **Vector construction** | |
| MyD88-KpnI-F | CGGGGTACCATGGCGTGTTGCGATAAATC |
| MyD88-XbaI-R | TGCTCTAGACATTGCCCAAACATTACAGG |
| MyD88-GFP-KpnI-F | CGGGGTACCATGGCGTGTTGCGATAAATC |
| MyD88-GFP-BamHI-R | CGCGGACGAGACATTGCCCAAACATTACAGG |
| IRF3-KpnI-F | CGGGGTACCATGTCTCATTCTAAACCTCTGCTCATC |
| IRF3-XbaI-R | TGCTCTAGAGTGTCAGTACAGCTCCATCATCTC |
| IRF3-dDBD-EcoRI-F | CCGGAATTCAACTCTAGTGCTGGATCC |
| IRF3-dDBD-EcoRI-R | CCGGAATTCAGCGTAATCTGGAACATCGT |
| IRF3-dIAD-EcoRI-F | CCGGAATTCCCAGACAACAGGCCTTGGGAG |
| IRF3-dIAD-EcoRI-R | CCGGAATTCATCTCCATCTCTGGTCTTGTT |
| IRF3-dSRD-EcoRI-F | CCGGAATTCCTCGAAGAGATGATGGAGCTG |
| IRF3-dSRD-EcoRI-R | CCGGAATTCGGCGCCGCCTCCAACAGCCA |
| IRF3-shRNA-F | GATCCGCTTCAAACTGGTCTCTGATTCAAGAGATCAGAGACCAGTTTGAAGCTTTTTTG |
| IRF3-shRNA-R | AATTCAAAAAAGCTTCAAACTGGTCTCTGATCTCTTGAATCAGAGACCAGTTTGAAGCG |
| IRF8-BamHI-F | CGCGGATCCATGTCAAACACGGGAGGTC |
| IRF8-XbaI-R | TGCTCTAGAAGTTCAGGCAGTGATTGGC |
| IRF8-dIRF-XhoI-F | CCGCTCGAGGAGGAGCAGAAGAATGGC |
| IRF8-dIRF-XhoI-R | CCGCTCGAGCCGACCTCCCGTGTTTGACAT |
| IRF8-dIRF3-XhoI-F | CCGCTCGAGGATGCTGTGAATATGCGC |
| IRF8-dIRF3-XhoI-R | CCGCTCGAGCATCATTTGGGAGAAAGC |
| IRF8-shRNA1-F | GATCCGCCGCACTTTGTTTCGAATTTCAAGAGAATTCGAAACAAAGTGCGGCTTTTTTG |
| IRF8-shRNA1-R | AATTCAAAAAAGCCGCACTTTGTTTCGAATTCTCTTGAAATTCGAAACAAAGTGCGGCG |
| **Real-time PCR** | |
| MyD88-RT-F | AGTTGGAACAGACCGAGTA |
| MyD88-RT-R | TGAGGAAGCGTAAGATGC |
| IRF3-RT-F | GAATGATGCTGCTAACCC |
| IRF3-RT-R | CGACTGGAGTCTCAAACG |
| IRF-8-RT-F | ACACGCAGGGAAACAGGATT |
| IRF-8-RT-R | GCACAGCGGAGTCTGGTCTT |
| β-actin-RT-F | GAGCCGCACGCTTCTTT |
| β-actin-RT-R | CTGCTGTAGCCGAGGAC |
| EF-1α-RT-1F | CGCCATCGTCAAACTC |
| EF-1α-RT-1R | CCACGGTCTGCCTCAT |
